# Mucosal Nanoemulsion Allergy Vaccine Suppresses Alarmin Expression and Induces Bystander Suppression of Reactivity to Multiple Food Allergens

**DOI:** 10.3389/fimmu.2021.599296

**Published:** 2021-02-25

**Authors:** Mohammad Farazuddin, Jeffrey J. Landers, Katarzyna W. Janczak, Hayley K. Lindsey, Fred D. Finkelman, James R. Baker, Jessica J. O’Konek

**Affiliations:** ^1^ Mary H. Weiser Food Allergy Center, University of Michigan, Ann Arbor, MI, United States; ^2^ Division of Allergy, Immunology and Rheumatology, University of Cincinnati College of Medicine, Cincinnati, OH, United States

**Keywords:** adjuvant, alarmins, allergy treatment, food allergy, immunotherapy vaccines and mechanisms, vaccine

## Abstract

We have demonstrated that intranasal immunotherapy with allergens formulated in a nanoemulsion (NE) mucosal adjuvant suppresses Th2/IgE-mediated allergic responses and protects from allergen challenge in murine food allergy models. Protection conferred by this therapy is associated with strong suppression of allergen specific Th2 cellular immunity and increased Th1 cytokines. Here we extend these studies to examine the effect of NE-allergen immunization in mice sensitized to multiple foods. Mice were sensitized to both egg and peanut and then received NE vaccine formulated with either one or both of these allergens. The animals were then subjected to oral challenges with either egg or peanut to assess reactivity. Immunization with NE formulations containing both egg and peanut markedly reduced reactivity after oral allergen challenge with either allergen. Interestingly, mice that received the vaccine containing only peanut also had reduced reactivity to challenge with egg. Protection from oral allergen challenge was achieved despite the persistence of allergen-specific IgE and was associated with strong suppression of both Th2-polarized immune responses, alarmins and type 2 innate lymphoid cells (ILC2). NE-induced bystander suppression of reactivity required IFN-γ and the presence of an allergen in the NE vaccine. These results demonstrate that anaphylactic reactions to food allergens can be suppressed using allergen-specific immunotherapy without having to eliminate allergen-specific IgE and suggests that modulation of Th2 immunity towards one allergen may induce bystander effects that suppress reactivity to other allergens through the induction of IFN-γ and suppression of alarmins in the intestine. In addition, these data suggest that a NE vaccine for a single food allergen may lead to a global suppression of allergic responses to multiple foods.

## Introduction

Food allergy is an emerging epidemic that now affects up to 15 million people in the US, including 8% of children. The economic burden of food allergy in the US alone exceeds $24.8 billion (1). Allergen-specific immunotherapy for food allergy involves the progressive administration of increasing amounts of a specific allergen by one of several routes and has been the primary approach to suppress allergic reactivity. This approach, however, does not provide long-term protection following cessation of therapy and requires prolonged treatment protocols burdening to patients and their families. Specifically, subcutaneous immunotherapy to food allergens showed promise for protection against IgE-mediated food allergies, however significant adverse reactions limited successful implementation (2–4). Sublingual, oral (OIT) and epicutaneous immunotherapy have demonstrated efficacy in animal models and human trials, however these approaches desensitize only a portion of patients and the protection achieved is rapidly lost after cessation of the therapy (5–11). Thus, there is a need to understand immune mechanisms that modulate Th2-biased immune responses to food and could lead to long-lasting protection from allergic reactions.

The primary immunologic mechanism of allergic hypersensitivity is the induction of Th2-polarized cellular immune responses leading to the production of allergen-specific IgE antibodies critical for mast cell activation. Th2 cytokines also are critical mediators of local allergic inflammation, including IL-4– and IL-13–dependent mucus production and IL-5–mediated eosinophil recruitment (12). Oral or subcutaneous allergen immunotherapy (AIT) appears to achieve desensitization to the allergen by temporarily reducing Th2-biased immunity and allergen-specific IgE. While OIT has been proven clinically useful for treating food allergy, it has not induced a long term redirection of allergen-specific immunity away from a Th2 phenotype (13). Thus, interest has been directed toward new strategies that are able to permanently suppress Th2 cellular immune responses or redirect these cellular Th2 responses towards a Th1 phenotype (14, 15).

We have developed a novel nasal vaccine-based immunotherapy system employing a nanoscale oil-in-water emulsion (nanoemulsion, NE) adjuvant. When administered intranasally (i.n.) with viral and bacterial antigens, this formulation induces robust systemic and mucosal immunity, and cell-mediated immune responses are polarized towards Th1 and Th17 (16–24). We have previously reported that therapeutic immunization with NE and antigen/allergen can suppress established Th2-polarized immunity and protect from allergen challenge in murine models of food allergy (25–28). NE-based allergy vaccines induced sustained unresponsiveness lasting at least 16 weeks, and protection was associated with increased IL-10 and regulatory T cells. Here, we extended our previous work to determine the ability of NE-based allergy vaccines to broadly suppress allergic reactions in mice sensitized to more than one food.

## Materials and Methods

### Antigen and Adjuvants

Nanoemulsion adjuvant (NE) was produced by a high speed emulsification of ultra-pure soybean oil with cetyl pyridinium chloride, Tween 80 and ethanol in water, with resultant NE droplets with average 350–400 nm diameter (17, 29). Aluminum hydroxide (alum, alhydrogel) was purchased from InvivoGen. Peanut extract (Greer) was used for all intraperitoneal (i.p.) and intranasal (i.n.) immunizations. For oral/intragastric (i.g.) challenges, peanut flour (12% fat, light roast, Byrd Mill) was solubilized in PBS. Endotoxin-free ovalbumin (OVA) was purchased from Hyglos. Endotoxin content of all vaccine components was determined by a limulus amebocyte lysate (LAL) assay (Pierce).

### Mice and Immunizations

**Figure 1:**
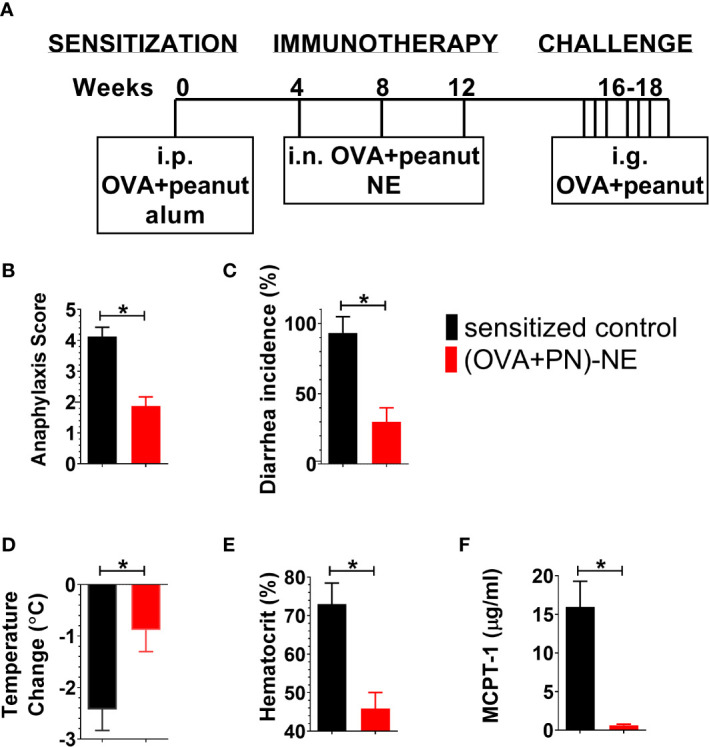
NE immunotherapy protects against allergic reactivity in polysensitized mice. **(A)** Mice were sensitized with OVA and peanut-alum and treated i.n. with 3 administrations of PBS (sensitized control) or OVA and peanut-NE (OVA+PN-NE) Mice were challenged orally with OVA and peanut and **(B)** symptoms of anaphylaxis, **(C)** diarrhea and **(D)** temperature change were monitored. **(E)** Hemoconcentration was determined by hematocrit. **(F)** Levels of MCPT-1 in the serum 60 min after challenge were determined by ELISA. Statistically significant differences (p < 0.05) are indicated by *.

Specific pathogen-free BALB/c mice (females 3 weeks old) were purchased from Jackson Laboratory. Mice were 4 weeks of age at the onset of the experiment. The experimental design is shown in **Figure 1**. In all experiments, allergic sensitization was induced with i.p. immunizations of 20 µg OVA and 20 µg peanut extract (PN) adsorbed on 1 mg alum at week 0. The experimental design for each experiment is as follows.

#### Assessment of Combination Vaccine Containing OVA and Peanut

Mice received i.n. immunizations of 12 µl (6 µl/nare) of a formulation containing 20 µg of OVA and 20 µg PN at weeks 4, 8 and 12. Sensitized control mice received i.n. PBS. Beginning 4 weeks after the final i.n. vaccine dose, anaphylaxis was induced by repeated oral challenge with allergen. Mice were challenged orally every other day for a total of 7 gavages (30). For each challenge, mice were fasted for 5–6 h to ensure gastric emptying and then were challenged by oral gavage (i.g.) with 0.2 ml containing of 10 mg OVA and 10 mg peanut. Reactivity was measured as described below.

#### Assessment of Bystander Protection

Mice received i.n. immunizations of a formulation containing either 20 µg of OVA or 20 µg PN mixed with 20% NE at weeks 4, 8 and 12. Mice were orally challenged with either 10 mg OVA or 10 mg peanut protein and reactivity was determined as described below.

#### Assessment of Requirement of Specific Antigen for Protection

Following the same schedule as described above, sensitized mice received i.n. immunizations of 20% NE alone (no antigen) or 20 µg of OVA in 20% NE. Sensitized control mice received i.n. PBS. Mice were challenged orally with OVA and reactivity was determined as described above. In a separate experiment following the same schedule, mice were immunized with 20 µg hepatitis B surface antigen (HBsAg) mixed with 20% NE.

#### IFN-γ Depletion Experiments

Mice were sensitized to OVA and peanut at week 0 and received i.n. PN-NE vaccine at weeks 4, 8 and 12. Mice were subjected to our repeated OVA challenge protocol beginning at week 16. Mice were injected i.p. with 0.5 mg anti-IFN-γ (XMG-6) or isotype control rat IgG1 (GL113) [both produced in house (31)] the day before starting oral challenges and every 4 days until the final challenge. All animal procedures were performed according to the National Institutes of Health guide for the care and use of laboratory animals and approved by the University of Michigan Institutional Animal Care and Use Committee (IACUC).

### Assessment of Hypersensitivity Reactions

Anaphylactic symptoms were evaluated for one hour following the final (7^th^) challenge with OVA using the following scoring system [modified from (32, 33)]: 0, no symptoms; 0.5, transient rubbing and scratching; 1, prolonged rubbing and scratching around the nose, eyes or head; 2, puffiness around the eyes or mouth, diarrhea, piloerection, and/or decreased activity with increased respiratory rate; 3, labored respiration, wheezing, stridor, and/or cyanosis around the mouth and tail; 4, tremor, convulsion, no activity after prodding and/or moribund; 5, death. Rectal temperature was monitored prior to and every 15 min for 60 min following challenge, and the maximum temperature change from baseline was reported. Mice were bled 60 min following challenge, and serum mouse mast cell protease-1 (MCPT-1) was determined by ELISA (eBioscience). To determine hemoconcentration, blood was drawn 60 min following challenge into heparinized capillary tubes and centrifuged for 5 min at 10,000 rpm. Hematocrit values were calculated as the length of packed RBCs as a percentage of the total length of serum and red cells in the capillary tube.

### Measurement of Serum IgE

Sera were obtained by cardiac puncture post-euthanasia one day after the final (7^th^) allergen challenge. Serum was separated from whole blood by centrifugation at 1,500×g for 5 min after allowing coagulation for 30–60 min at room temperature. Serum samples were stored at −20°C until analyzed. OVA-specific IgE antibody levels were determined by ELISA. Serially diluted serum samples were incubated on microtiter plates coated with 20 µg/ml OVA. IgE antibodies were detected with alkaline phosphatase conjugated anti‐mouse IgE (Rockland) and Sigma Fast™ p‐nitrophenyl phosphate substrate and quantified by measuring the optical density (OD) at 405 nm. The antibody concentrations are presented as endpoint titers defined as the reciprocal of the highest serum dilution producing an OD above background of naïve sera. The cutoff value is determined as the OD (mean+2 standard deviations) of the corresponding dilution of naive sera (34, 35).

### Analysis of Cytokine Production

Mice were sacrificed one day after the final (7^th^) oral challenge, and mesenteric lymph nodes were harvested. The cellular recall response was evaluated in lymphocytes isolated from mesenteric lymph nodes. Single cell lymphocyte suspensions were cultured *ex vivo* ± OVA (20 µg/ml) at 37°C. After 72 h, cytokine secretion was measured in cell culture supernatants using Luminex Multiplex detection system (Millipore). For real-time PCR analysis, RNA was isolated from duodenum homogenates with an RNeasy mini kit (Qiagen), and cDNA was generated with a Superscript II reverse transcription kit (Invitrogen). qPCR was performed with SYBR green master mix and commercially available primer sets (Bio-Rad). Values were normalized to GAPDH and displayed as fold induction over control samples.

### Lamina Propria Mononuclear Cells Isolation

Mice were sacrificed one day after the final (7^th^) oral challenge. Small intestine (SI, 15 cm) was dissected from the mouse and Peyer’s patches were trimmed off. SI was cut longitudinally and washed with PBS thoroughly to remove ingested food. SI was incubated in a petri dish with 10 ml PBS with EDTA (5 mM) for 10 min on ice. Intestines were washed with PBS (no EDTA) by vortexing vigorously to remove the epithelial cells. These two steps were repeated 3–4 times until the tissue became clear. Tissue was minced finely and transferred to 8 ml digestion buffer (16 mg collagenase A (Roche) and 1.6mg DNase I (Roche) in RPMI (10% FBS) and incubated at 37°C for 30 min. After incubation, digested tissue was passed through a 10 ml syringe with 18G needle a few times. Liberated cells were filtered through 70 µm filter. The cell suspension was washed by adding 20 ml of RPMI with 10% FBS. The cell pellet was suspended in 44% Percoll (4 ml) and loaded on 67% Percoll (3 ml) for centrifugation. A mononuclear cell gradient was created by spinning the cells down at 1,800 rpm for 20 min at room temperature with centrifuge acceleration set at 5 and deceleration set to 0. The middle interphase of mononuclear cells was collected from the interface and washed again with RPMI (10% FBS). The obtained cells were counted and used for subsequent analysis.

### Antibodies

All the antibodies used for flow cytometry were purchased from eBioscience, Biolegend and BD biosciences. For cell surface staining, a lineage cocktail consisting anti-mouse CD3 (clone 145-2C11), anti-mouse Ly-6G/Ly-6C (clone RB6-8C5), anti-mouse CD11b (clone M1/70), anti-mouse CD45R/B220 (clone RA3-6B2), and anti-mouse TER-119/Erythroid cells (clone Ter-119) was used. FITC-streptavidin was used to stain biotin labelled primary antibody cocktail. Other antibodies used were rat anti-mouse CD45, anti-mouse CD127 (clone A7R34), anti-mouse CD90.2 (clone 53-2.1), anti-mouse KLRG1 (clone 2F1), and anti-mouse GATA3 (clone TWAJ). Foxp3 fixation and permeabilization kit (eBioscience) was used for intracellular staining.

### Flow Cytometry

Cells were stained with lineage antibody cocktail on ice for 20 min followed by washing with flow staining buffer (PBS with 0.1% BSA) two times. Cells were then incubated with FITC-streptavidin antibody on ice for another 20 min. Cells were washed two times with flow staining buffer. Cells were then stained with live-dead ef450, CD45, CD127, CD90.2, and KLRG1 on ice for 20 min followed by washing two times with flow staining buffer. Intracellular staining of GATA3 was done using Foxp3 fixation and permeabilization kit as per the manufacturer’s protocol (eBiosciences). Samples were acquired on Novocyte 3000 (Acea biosciences) and data were analyzed using FlowJo v10.1. The gating strategy for identifying ILC2s is shown in **Supplementary Figure 1**.

### Statistics

Results presented here are the representatives of at least two independent experiments. Each experiment contained 8–10 mice per group. Statistical comparisons were assessed by the Mann-Whitney test using GraphPad Prism version 8 (GraphPad Software). The *p* value < 0.05 was considered as significant.

## Results

### Intranasal Immunization With Allergens in NE Adjuvant Suppresses Allergic Reactions in Polysensitized Mice

BALB/c mice were sensitized to egg and peanut and immunized with OVA and peanut formulated in NE to determine if suppression of reactivity to 2 allergens could be achieved simultaneously. Sensitized control mice had profound physiological reactions to challenge as indicated by severe symptoms of anaphylactic shock, including diarrhea, labored respiration, wheezing, lack of activity when prodded, core body temperature loss of greater than 2°C, hemoconcentration and increased mast cell degranulation (MCPT-1) (**Figure 1**). The NE vaccine markedly suppressed these responses to allergen challenge. Anaphylaxis symptoms were markedly reduced to mild symptoms such as pruritus or reduced activity (**Figure 1B**), and the incidence of diarrhea was reduced from 100–40% (**Figure 1C**). Mice treated with the NE vaccine also were protected from hypovolemic shock and experienced minimal body temperature loss while hemoconcentration also was prevented (**Figures 1D, E**). MCPT-1 measured in serum following challenge was used to assess mast cell degranulation. Consistent with the clinical symptoms of allergic reaction, immunized mice had significant reductions in MCPT-1, with average levels of 0.6 µg/ml compared with 16 µg/ml in sensitized control mice (**Figure 1F**; p=0.0079).

### Immunization of Polysensitized Mice With NE Adjuvant Induces Bystander Suppression of Allergic Reactivity

**Figure 2:**
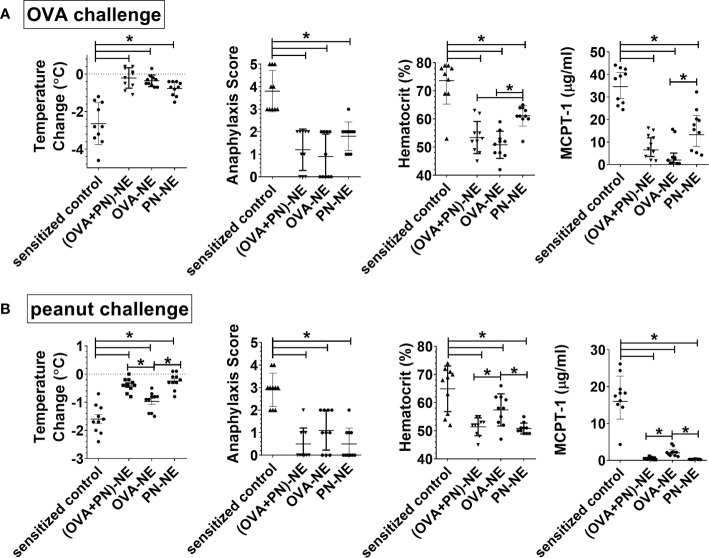
Immunization of polysensitized mice with NE and one allergen provides protection against reactivity to another allergen. As described in Figure 1A, mice were sensitized with OVA and peanut-alum and treated i.n. with 3 administrations of PBS (sensitized control), ova and peanut-NE (OVA+PN-NE), OVA-NE or peanut-NE (PN-NE). Mice were challenged orally with **(A)** OVA or **(B)** peanut and temperature change and symptoms of anaphylaxis were monitored. Hemoconcentration was determined by hematocrit. Levels of MCPT-1 in the serum 60 min after challenge were determined by ELISA. Statistically significant differences (p < 0.05) are indicated by *.

Next, we wanted to assess the effects of immunotherapy with NE and only one allergen on protection in polysensitized animals. Mice were again sensitized to both egg and peanut, and then were nasally treated with either OVA or peanut or both allergens formulated in NE. In general, mice were protected from allergic reaction to whatever allergen was contained in the vaccines, and protection for each allergen was similar if the mice were treated with the vaccine containing either a single allergen or both allergens (**Figure 2**). Surprisingly, mice that were treated with the OVA-NE vaccine were also protected from reactivity to challenge with peanut and mice treated with the PN-NE vaccine were protected from challenge with OVA. While there was a trend that this “bystander protection” was not as complete as protection induced by immunotherapy with NE and both allergens, these differences were not significant, and mice immunized with only one allergen in NE had significantly less severe allergic reactions compared with sensitized control mice that did not receive the i.n. vaccine. Bystander suppression of reactivity persisted for at least 8 weeks after the final vaccine dose (**Supplementary Figure 2**).

**Figure 3:**
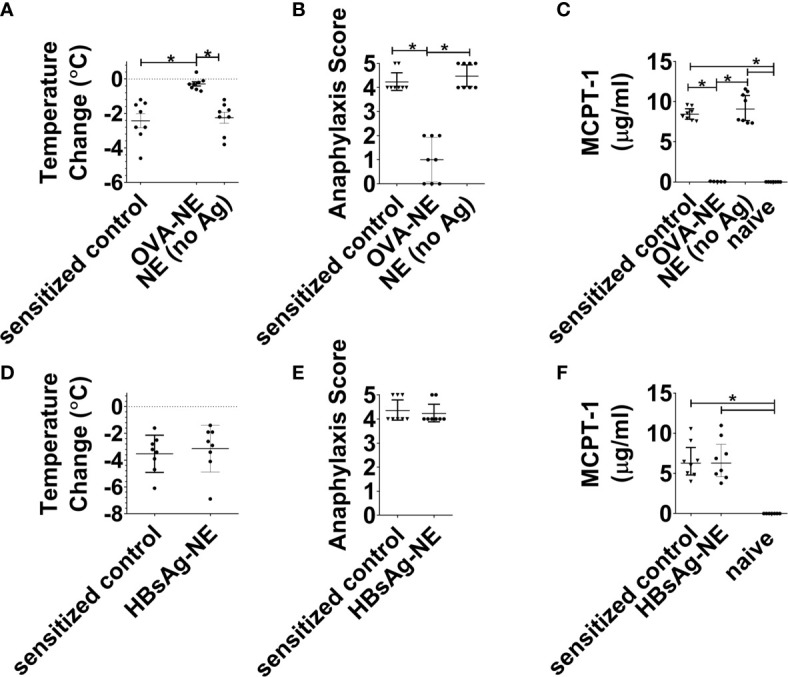
Intranasal administration of NE without allergen does not suppress the allergic response. **(A–C)** Mice were sensitized with OVA and peanut-alum and treated i.n. with 3 administrations of PBS (sensitized control), OVA-NE (OVA-NE) or NE only (no antigen) or Mice were challenged orally with OVA and temperature change and symptoms of anaphylaxis were monitored. Serum MCPT-1 levels were determined by ELISA. **(D–F)** In a separate experiment, mice were similarly sensitized with OVA and peanut with alum and treated i.n. with 3 administrations of PBS (sensitized control) or hepatitis B surface antigen-NE (HBsAg-NE). Statistically significant differences (p < 0.05) are indicated by *.

We next aimed to determine if the observed bystander suppression of allergic reactivity required immunotherapy with NE and at least one allergen to which the mice were sensitized. As shown in **Figures 3A–C**, in mice that were sensitized to OVA and peanut, i.n. instillation of NE alone (no allergen) did not induce any suppression of allergic reactivity to oral OVA challenge, as reactivity in these mice was equivalent to sensitized control mice and significantly more severe than mice that received the OVA-NE vaccines. Similarly, NE formulated with an unrelated antigen [hepatitis B surface antigen (HBsAg)] did not confer protection from challenge with OVA (**Figures 3D–F**).

### Intranasal Immunization With NE Adjuvant Suppresses Allergy Associated Th2 Responses and Alarmin Expression

**Figure 4:**
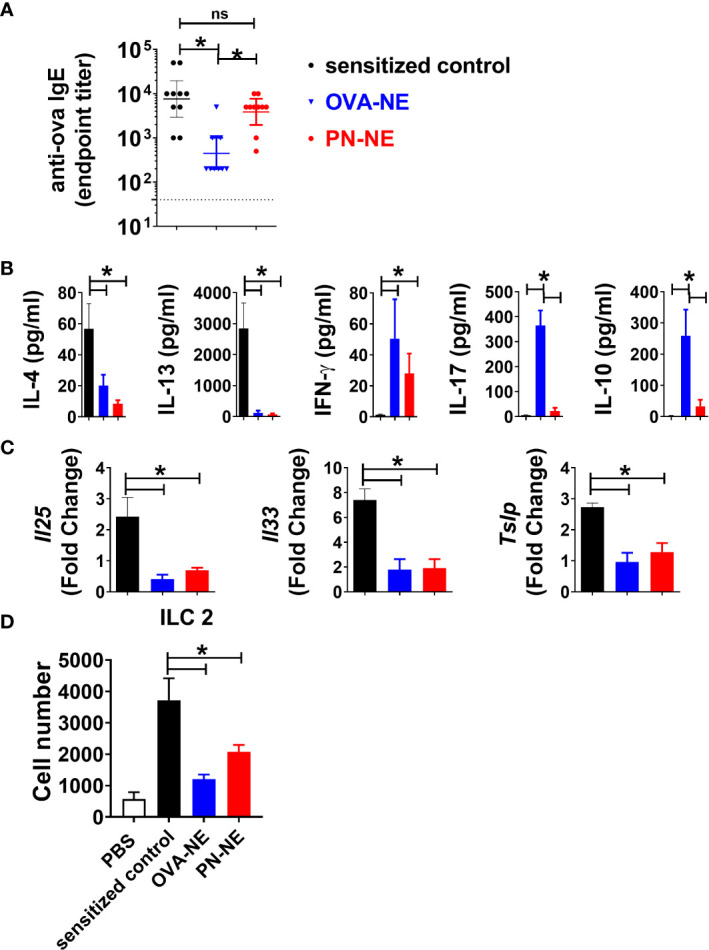
NE immunotherapy induces antigen-specific and bystander suppression of Th2 cytokines and alarmins. As described in **Figure 1A**, mice were sensitized with OVA and peanut-alum and treated i.n. with 3 administrations of PBS (sensitized control), OVA-NE or peanut-NE (PN-NE). Mice were challenged orally with OVA, and **(A)** OVA-specific IgE was measured in serum obtained after the final challenge and **(B)** OVA-specific cytokine secretion was determined in cultures of mLN lymphocytes. **(C)** Duodenum samples were homogenized and mRNA extracted to determine relative gene expression compared to GAPDH. **(D)** Total number of ILC2 (Lin- CD45+ CD127+ CD90.2+ KLRG1+ GATA3+) from SI. Statistically significant differences (p < 0.05) are indicated by *.

While food allergic reactions are dependent upon the presence of allergen-specific IgE, many patients with allergen-specific IgE to foods do not clinically react to those foods. In the present study, immunization with PN-NE suppressed allergic reactivity to both peanut and OVA without significantly reducing OVA-specific IgE (**Figure 4A**). This apparent disconnect between the presence of allergen-specific IgE and reactivity to an allergen suggests that other immune changes are behind the suppression of allergic reactions observed here.

Allergen-specific cytokine secretion was measured to characterize changes to the Th2-biased cellular immune responses associated with allergic disease. Upon stimulation with OVA, cells from (OVA and peanut)-alum sensitized mice produced significant levels of Th2-type cytokines IL-4 and IL-13 but not IFN-γ (**Figure 4B**). Immunotherapy with either OVA or peanut in NE reduced OVA-specific IL-4 and IL-13 and increased IFN-γ. While OVA-specific IL-17 and IL-10 were increased in mice that received the OVA-NE vaccine, the PN-NE vaccine did not affect OVA-specific production of these cytokines. Because of the interplay between Th2 immunity and expression of alarmins, expression of the genes for the alarmins IL-25, IL-33, and TSLP was also evaluated in the small intestine (36–40). Immunization with the NE vaccines significantly reduced the expression of *Il25*, *Il33* and *Tslp* such that the fold change over expression in naïve mice was approximately 1 (**Figure 4C**). These data indicate that immunization with NE prevents increased alarmin expression normally observed in allergen sensitized mice.

### Intranasal Immunization With NE Adjuvant Suppresses ILC2 Populations

We observed a significant decrease in alarmin expression, in animals that received the NE-allergen vaccines compared to sensitized control animals. It has been previously established that IL-25 and IL-33 acts as activation cytokines and regulate ILC2 populations in allergic inflammation (41–43). ILC2s were quantified in the small intestine lamina propria to determine if reduction in reactivity was associated with reduce ILC2 accumulation in the tissue. ILC2s were increased in the intestine of sensitized mice compared to naïve. Conversely, ILC2s were significantly reduced in OVA-NE-and PN-NE immunized animals compared to sensitized controls (**Figure 4D**). These data suggest that immunization with either OVA-NE-or PN-NE modulates epithelial alarmin production, which in turn prevents the accumulation of ILC2s in the tissues.

### Bystander Protection Induced by NE Allergy Vaccines Requires IFN-γ

**Figure 5:**
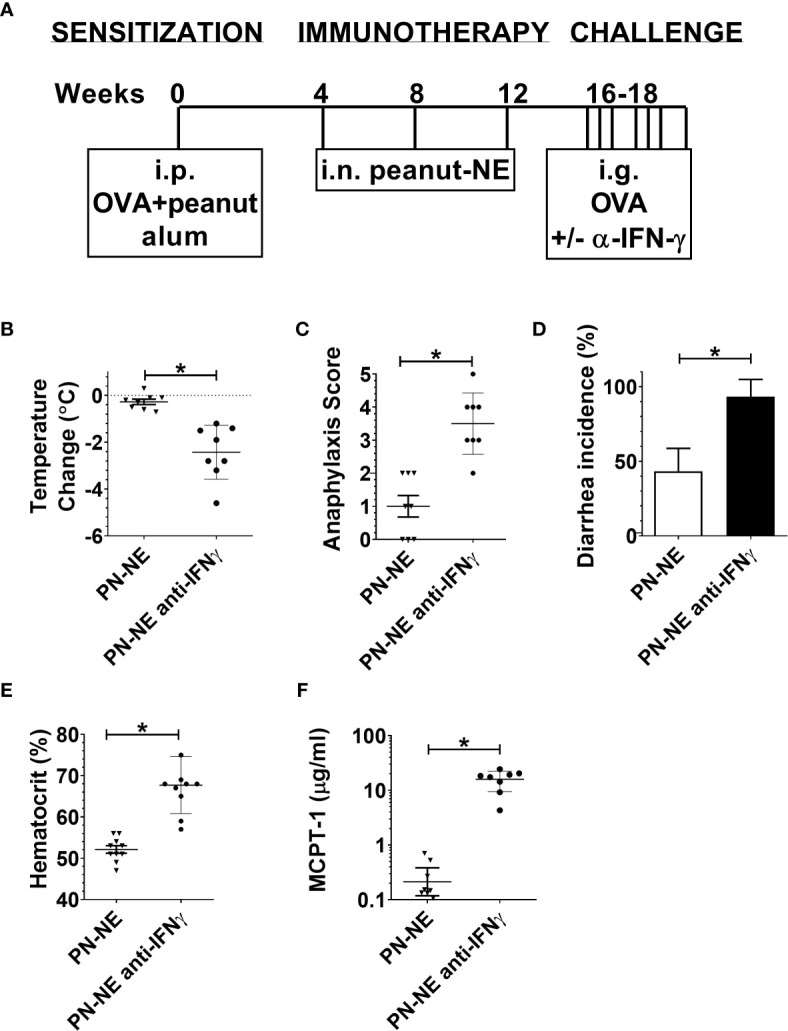
Bystander protection induced by NE allergy vaccines requires IFN-γ **(A)** Mice were sensitized with OVA and peanut-alum and treated i.n. with 3 administrations of PBS (sensitized control) or peanut-NE (PN-NE). Mice were challenged orally with OVA and IFN- **γ** was depleted during the challenge phase. **(B)** Temperature change, **(C)** symptoms of anaphylaxis and **(D)** diarrhea were monitored. **(E)** Hemoconcentration was determined by hematocrit. **(F)** Levels of MCPT-1 in the serum 60 min after challenge were determined by ELISA. Statistically significant differences (p < 0.05) are indicated by *.

**Figure 6:**
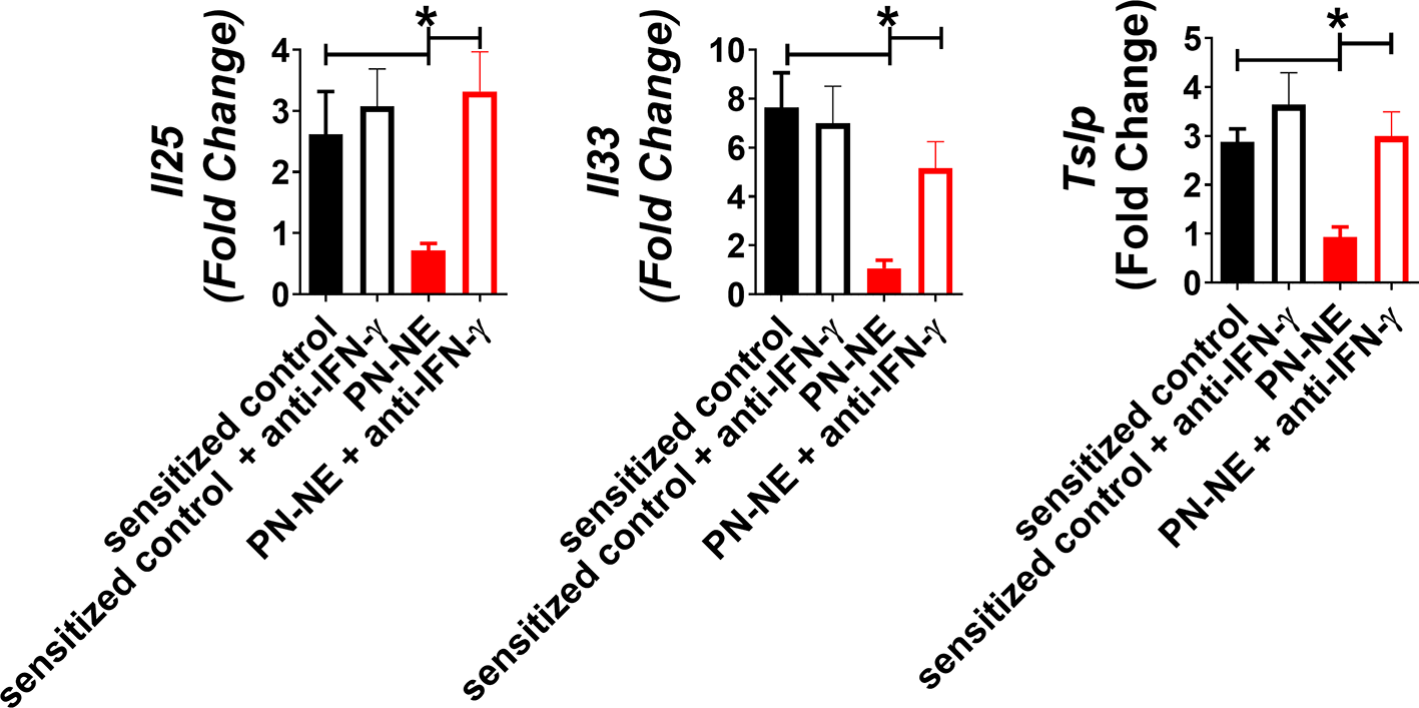
IFN-γ is required for suppression of alarmins by NE allergy vaccines. Mice were sensitized with OVA and peanut-alum and treated i.n. with 3 administrations of PBS (sensitized control) or peanut-NE (PN-NE). Mice were challenged orally with OVA and IFN- **γ** was depleted during the challenge phase. Duodenum samples were homogenized and mRNA was extracted to determine relative gene expression compared to GAPDH. Statistically significant differences (p < 0.05) are indicated by *.

Because NE immunization increased IFN-γ, which has been shown to suppress Th2 immunity and alarmins, we hypothesized that bystander protection associated with NE was IFN-γ dependent. Mice were sensitized to OVA and peanut and then treated with PN-NE. During the allergen challenge phase in which mice were treated with OVA, IFN-γ was depleted. Depletion of IFN-γ during the challenge phase completely abrogated the protection induced by the PN-NE vaccine, as mice treated with anti-IFN-γ antibody had severe reactions to challenge, including decreases in core body temperature and increased clinical symptoms, diarrhea, hematocrit and MCPT-1 similar to animals not treated with NE (**Figure 5**). Suppression of alarmin expression by the PN-NE vaccine was also reversed following IFN-γ depletion, as mice that were depleted of IFN-γ had similar expression of alarmins in the small intestine as sensitize mice that did not receive the vaccine (**Figure 6**).

## Discussion

Thirty to forty percent of patients with food allergies are sensitized to multiple foods (44, 45). While allergen-specific immunotherapy has the potential to relieve the burden of fear of reactivity to specific foods, this approach is more difficult for polysensitized individuals. Approved, allergen-specific immunotherapy for food allergy involves a single food, and regulatory issues may preclude the development of therapies containing multiple foods. While some studies have demonstrated the ability to desensitize patients with OIT for up to 5 foods simultaneously (multi-OIT) (46, 47), the amount of each food required to be consumed daily is a burden for some children as the food required for multi-OIT can be a significant proportion of the daily caloric intake for a child. Given this, there is interest in the development of therapies for food allergy that work more broadly and are not specific to one allergen.

The present studies demonstrate that NE can be formulated with multiple allergens and lead to reduction of allergic reactivity to all allergens included in the vaccine. NE was formulated with two allergens and maintained therapeutic efficacy for both foods. Based on the protein loading capacity of NE, formulation with more than 2 allergens would also be possible and the therapy only has to be administered three to four times to achieve sustained unresponsiveness of at least 4 months (25–27). Given that long-term protection can be achieved with only a few doses administered at monthly intervals, this approach would significantly reduce the burden on patients with multiple food allergies over daily, allergen-specific immunotherapies.

Importantly, these studies also demonstrate a “bystander” effect in that NE-induced reduction of allergic reactivity with one allergen also reducing reactions to unrelated allergens not included in the vaccine. The bystander protection did require immunization with an allergen to which the animal was previously sensitized, suggesting the non-specific reduction of food allergic reactions required prior sensitization and immune recognition of the vaccine. This bystander suppression of reactivity was maintained for at least 8 weeks, supporting our previous work that demonstrated the long-term sustained unresponsiveness induced by this approach (27). The mechanism of this effect appeared to be redirection of the underlying immune polarization from a Th2 to a Th1 phenotype, especially since no suppressive activity was demonstrated with treatment using either the adjuvant alone or with an immunogen for which there was no pre-existing allergy (HBsAg). This demonstrates that while NE allergy vaccines provide bystander protection, it requires the recognition and induction of an antigen-specific immune response.

There are very few reports of bystander activation of antigen non-specific immune responses, but there is some evidence these do exist. Immunization with aluminum adjuvants (alum) has been shown to induce bystander polarization of Th2 immune responses to unrelated antigens. Specifically, if mice were immunized with alum and “antigen 1”, and then later exposed to antigens 1 and 2 together in the absence of alum, mice developed Th2-polarized immune responses to antigen 2 (48, 49). These studies demonstrated the need for co-administration of antigens to drive the bystander effects. Eisenbarth et al. described this phenomenon as “collateral priming” and demonstrated that it was based on an adaptive T cell-derived, cytokine dependent mechanism that did not require innate toll-like receptor 4 signaling (49).

Other studies also support the concept of protection to proteins not included in the immunotherapy. For example, after sensitization with whole peanut extract, immunotherapy with individual peanut components such as Ara h 1 or Ara h 2 conferred protection against challenge with the whole allergen, despite the fact that mice were sensitized to multiple protein components of the allergen that the immunotherapy did not target (50–52). It also has been reported that epicutaneous immunotherapy with peanut in mice generates a Treg population that can prevent the subsequent sensitization to either peanut or house dust mite (53, 54). These Tregs were broadly effective at suppressing inflammation, including T cell-mediated intestinal inflammation in a mouse model of colitis (55). Along with our data, these reports suggest that antigen-specific T cell responses to newly introduced immunogens can be influenced by non-specific elements (mainly regulatory cells and cytokines) from ongoing immune responses to other antigens.

While our previous work has shown that induction of Tregs and IL-10 were important to NE mediated immune modulation (25, 28), we now include the production of IFN-γ as critical for allergic protection. Interestingly, while the NE vaccines induced antigen-specific production of IL-17 and IL-10, there was no bystander modulation of these cytokines, as immunization with PN-NE did not induce production of OVA-specific IL-17 or IL-10. The significant increase in OVA-specific IFN-γ production in PN-NE-immunized mice suggested a key role of IFN-γ in driving the bystander modulation of allergen-specific immune responses and reduction of allergic reactivity. The induction of Th1 cytokines, including IFN-γ, has been associated with resolution of food allergy and favorable outcomes for immunotherapy in humans (56). It has been suggested that IFN-γ can reduce allergic disease through suppression of Th2 cells as well as effects on the innate cells and alarmins which are required for both the induction and maintenance of allergic disease. Innate cytokines including the alarmins IL-25, IL-33, and TSLP are produced by epithelial cells and are key mediators of allergic disease (57). The NE vaccines in our studies suppressed alarmin expression in an IFN-γ dependent mechanism, and this correlated with reduction of allergic reactivity to bystander allergens. This suggests local NE-induced IFN-γ modulates the small intestine environment to suppress the allergic response. Since these components of the innate immune system function in an antigen-independent manner, this may be responsible for the non-antigen specific bystander effects that confer protection against allergens not included in the NE vaccine. Additional studies are required to determine if modulation of a specific alarmin is required for suppression of reactivity, and further dissection of the mechanism of IFN-γ induction on suppression of reactivity is currently in progress.

A role for IFN-γ–mediated suppression of ILC2s in the small intestine cannot be ruled out, as mice that received the NE vaccines also had reduced ILC2s in the small intestine. Previous work has demonstrated that in the lung, IFN-γ prevents the accumulation of these lymphoid cells in mucosal tissues by limiting the recruitment and maintenance of these cells (58–60). The reduction in ILC2s in the mice that received the NE vaccines may also be a critical factor for the observed reduction in allergic reactivity, as IL-4 and IL-13 producing ILC2s have also been shown to promote experimental food allergy (36, 61).

Overall, these results show that modulation of Th2 immunity towards one food can induce bystander effects that suppress allergic reactivity to unrelated foods. This may lead to a global reduction of allergic reactivity to multiple foods. This allergen-non-specific protection may also be induced by other therapies that increase IFN-γ and decreased expression of alarmins in the gut mucosa. Taken together, these results suggest new targets for the suppression of allergic disease.

## Data Availability Statement

The raw data supporting the conclusions of this article will be made available by the authors, without undue reservation.

## Ethics Statement

The animal studies were reviewed by the University of Michigan Institutional Animal Care and Use Committee.

## Author Contributions

MF, FF, JB, and JO designed the studies and prepared the manuscript. MF, JL, KJ, HL, and JO performed experiments and analyzed data. All authors contributed to the article and approved the submitted version.

## Funding

This project has been funded by a Food Allergy Research and Education New Investigator Award, the National Institute for Allergy and Infectious Disease, National Institutes of Health under Grant R01AI145991, the Michigan Food Allergy Research Accelerator (M-FARA), and a generous gift from Robert and Caren Vondell.

## Conflict of Interest

JB holds stock in Blue Willow Biologics, a company that has licensed the adjuvant technology from the University of Michigan. JB and JO’K are inventors of the adjuvant technology involved in this research and patent applications have been submitted for this technology.

The remaining authors declare that the research was conducted in the absence of any commercial or financial relationships that could be construed as a potential conflict of interest.
