# Supplementary material for: Mucosal Nanoemulsion Allergy Vaccine Suppresses Alarmin Expression and Induces Bystander Suppression of Reactivity to Multiple Food Allergens
Source: Front Immunol. 2021 Feb 25;12:599296. doi: 10.3389/fimmu.2021.599296 (PMC7946984; doi:10.3389/fimmu.2021.599296)
Supplement: Supplementary file 1 [file DataSheet_1.docx]

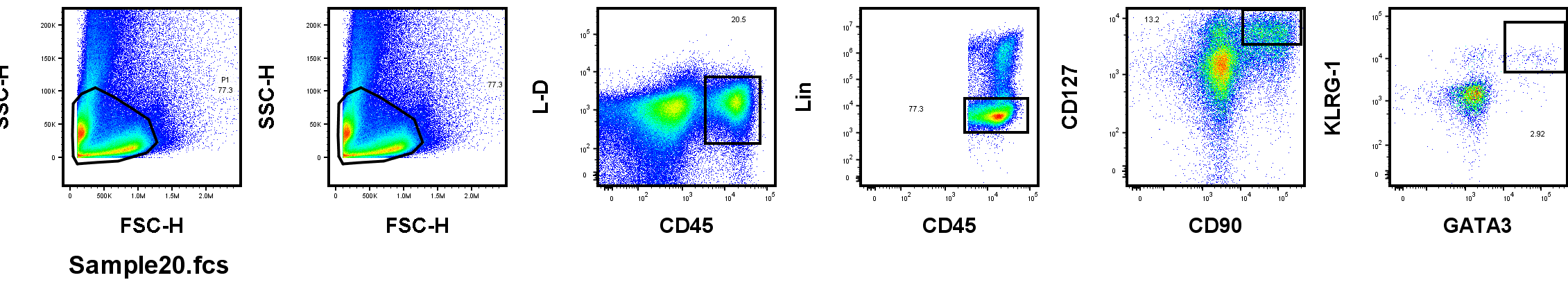


**Gating strategy for ILC2**

**(A) (B) (C) (D) (E)**

**Supplementary Figure 1. Gating strategy for analysis of ILC2 cells from small intestine.** As described in method section, SI cells were stained with lineage antibody cocktail, cell surface markers for ILC2 and transcription factor GATA3. Briefly, live CD45+ cells were gated (gate B). Further, lineage negative CD45+ cells (gate C) were gated for CD127+ and CD90+ cells (gate D). This total ILC population was gated as KLRG1 and GATA3 double positive ILC2 cell population (gate E).

 **Supplementary Figure 2. Immunization of polysensitized mice with NE and one allergen provides durable protection against reactivity to another allergen.** Mice were sensitized with OVA and peanut-alum and treated i.n. with 3 administrations of PBS (sensitized control), OVA and peanut-NE (OVA+PN-NE), OVA-NE or peanut-NE (PN-NE). Beginning 8 weeks after the final i.n. vaccine dose, mice were challenged orally with OVA and **(A)** temperature change was monitored. **(B)** Levels of MCPT-1 in the serum 60 min after challenge were determined by ELISA. Statistically significant differences (p<0.05) are indicated by *.
